# Unexpected Racemization in the Course of the Acetalization of (+)-(*S*)-5-Methyl-Wieland–Miescher Ketone with 1,2-Ethanediol and TsOH under Classical Experimental Conditions

**DOI:** 10.3390/ijms20246147

**Published:** 2019-12-05

**Authors:** Francesca Leonelli, Irene Piergentili, Giulio Lucarelli, Luisa Maria Migneco, Rinaldo Marini Bettolo

**Affiliations:** 1 Dipartimento di Biologia Ambientale, Università degli Studi La Sapienza, Piazzale Aldo Moro 5, I-00185 Roma, Italy; 2 Dipartimento di Chimica, Università degli Studi La Sapienza, Piazzale Aldo Moro 5, I-00185 Roma, Italy; irene.piergentili@gmail.com (I.P.); giulio.lucarelli@uniroma1.it (G.L.); luisamaria.migneco@uniroma1.it (L.M.M.); rinaldo.marinibettolo@uniroma1.it (R.M.B.)

**Keywords:** chiral synthons, (+)-(*S*)-5-methyl-Wieland–Miescher ketone, synthesis, chirality, acetalization

## Abstract

(+)-(*S*) and (−)-(*R*)-5-methyl-Wieland-Miescher ketone (+)-**1** and (−)-**1**, are important synthons in the diastereo and enantioselective syntheses of biological and/or pharmacological interesting compounds. A key step in these syntheses is the chemoselective C(1)O acetalization to (+)-**5** and (−)-**5**, respectively. Various procedures for this transformation have been described in the literature. Among them, the classical procedure based on the use of 1,2-ethanediol and TsOH in refluxing benzene in the presence of a Dean-Stark apparatus. Within our work on bioactive natural products, it occurred to us to observe the partial racemization of (+)-**5** in the course of the acetalization of (+)-**1** by means of the latter methodology. Aiming to investigate this drawback, which, to our best knowledge, has no precedents in the literature, we acetalized with 1,2-ethanediol and TsOH in refluxing benzene and in the presence of a Dean–Stark apparatus under various experimental conditions, enantiomerically pure (+)-**1**. It was found that the extent of racemization depends on the TsOH/(+)-**1** and 1,2-ethanediol/(+)-**1** ratios. Mechanism hypotheses for this partial and unexpected racemization are provided.

## 1. Introduction

**Figure 1:**
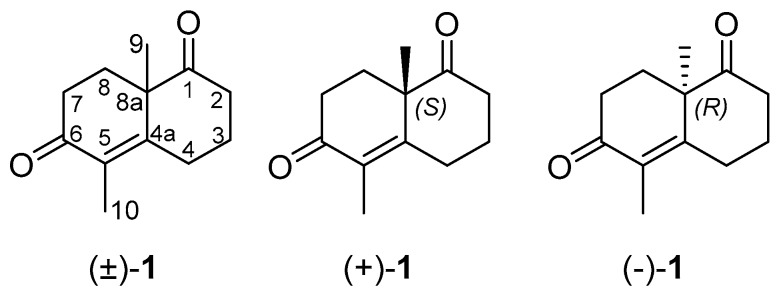
(±)-(*RS*)- and (+)-(*S*)- and (−)-(*R*)-5-methyl-Wieland-Miescher ketones, (±)-**1**, (+)-**1**, and (‒)-**1**.

The (±)-(*RS*)- and (+)-(*S*)- and (‒)-(*R*)-5-methyl-Wieland-Miescher ketones ((±)-(*RS*)-, (+)-(*S*)- and (‒)-(*R*)-5,8a-dimethyl-3,4,8,8a-tetrahydro-naphtalene-1,6(2*H*,7*H*)-diones) ((±)-**1** [1,2,3,4,5,6,7,8,9,10,11,12,13,14,15,16,17,18,19,20,21,22,23,24], (+)-**1**, and (‒)-**1** [25,26,27,28,29,30,31,32,33,34,35,36,37,38,39,40,41,42,43,44,45,46,47,48,49,50,51,52,53,54,55,56,57,58,59,60,61,62,63,64,65,66,67,68,69,70,71,72,73,74,75,76,77,78,79,80,81,82,83,84,85,86,87,88,89,90,91], Figure 1) are important synthons in the diastereo and enantioselective synthesis of biological and/or pharmacological interesting compounds.

**Scheme 1:**
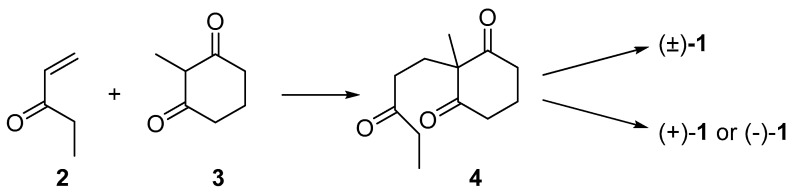
Preparation of (±)-**1**, (−)-**1** and (+)-**1**.

Racemate (±)-**1** can be obtained by the Robinson annulation of ethyl vinyl ketone **2** with 2-methyl-1,3-cyclohexanedione **3** (Scheme 1) [1,2,3,4,5,6,7,8,9,10,11,12,15,18,19,21,22,23,24,92,93,94,95,96,97]. This process does not require the isolation of intermediate **4**. The preparation of enantiomers (+)-**1** and (‒)-**1** was achieved by carrying out the cyclization of prochiral trione **4** in the presence of l- or d-amino acids (Scheme 1) [25,26,27,28,29,30,31,32,33,34,35,36,37,38,39,41,42,43,44,45,46,47,48,49,52,53,54,55,56,57,58,59,60,61,62,63,64,65,66,67,68,69,70,71,72,73,74,75,76,77,78,79,80,81,82,83,84,85,86,87,88,95,98,99,100,101,102,103,104,105,106,107,108,109].

Compounds **1** are versatile synthons because the α,β-unsaturated system allows the installment of the required stereochemistry at the ring junction and the introduction by reductive alkylation (from the α face, owing to the CH_3_-C(8a) steric hindrance) of a substituent at C(5). Afterward, the C(1)O can be exploited for further annulation processes. These synthetic operations are allowed by the different reactivity of C(1)O and C(6)O since the former can be chemoselectively acetalized. 

**Scheme 2:**
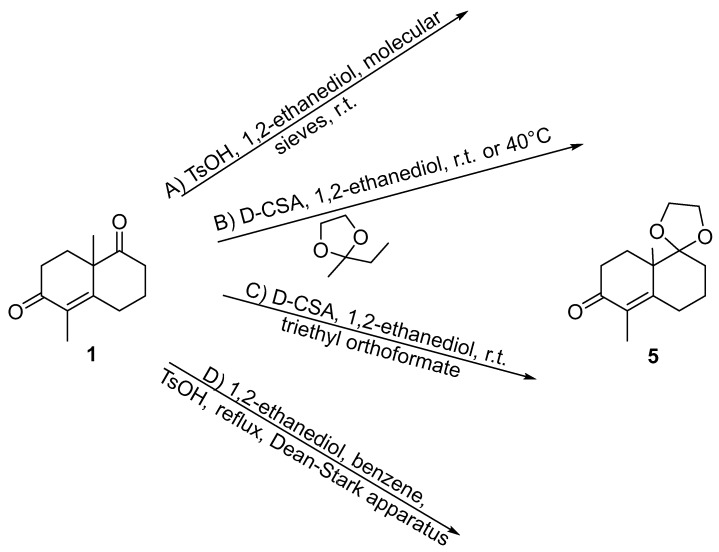
Acetalization of compound **1** carried out under different conditions as reported in the literature.

Various procedures (Scheme 2) for the chemoselective protection of C(1)O have been described in the literature, some of which lower the reaction temperature and increase the 1,2-ethanediol quantity, thus avoiding the protection of both carbonyl groups (conditions A [21,23,58,80,83,85,86,102], B [27,28,29,31,32,34,41,42,43,45,46,52,54,57,59,60,61,63,64,65,66,67,68,71,73,74,76,82,110] or C [109,111] in Scheme 2), while others, based on the use of 1,2-ethanediol and TsOH in refluxing benzene and in the presence of a Dean–Stark apparatus, require generally shorter reaction times [2,3,6,9,11,13,17,22,33,35,39,40,44,48,49,72,77,81,84,87,88,89,90,91,94,112,113,114,115,116] (D in Scheme 2). 

In the course of our work on bioactive natural products and bioactive materials [117,118,119,120,121,122,123,124], it occurred to us to observe the partial racemization of (+)-**5** during the acetalization of (+)-**1** with 1,2-ethanediol and TsOH in the presence of a Dean–Stark apparatus. According to our best knowledge, obtaining partially racemized (+)-**5** or (−)-**5** when the acetalization reaction is carried out by this procedure on (+)-**1** or (‒)-**1** [33,35,39,40,44,48,49,72,77,81,84,87,88,89,90,91,116], respectively, has not been previously reported in the literature, which was the reason behind investigating this reaction.

## 2. Results and Discussion

**Scheme 3:**
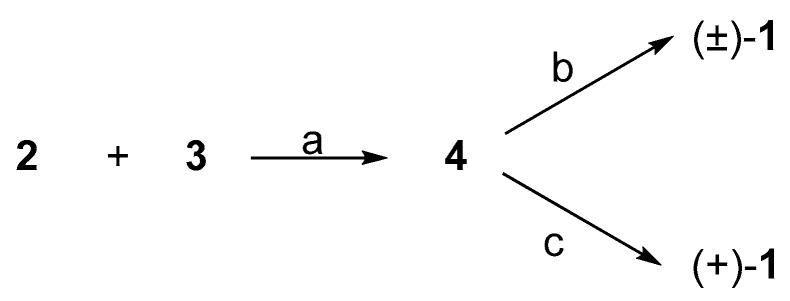
Preparation of (±)-**1** and (+)-**1**. Reaction conditions: a—tetrahydrofuran (THF), triethylamine (NEt_3_), reflux; b—toluene, pyrrolidine, reflux, 30% yield; c—dimethylsulfoxide (DMSO), l-phenylalanine, 1 M HClO_4_, 90 °C, 80% yield, enantiomeric excess (ee) from 87% to 96% after three crystallizations from *n*-hexane.

Enedione **1** was synthesized both in racemic and in the enantiomerically pure *S* form according to known methods (Scheme 3). The preparation of (±)-**1** was carried out in two steps where ethyl vinyl ketone **2** was reacted with 2-methyl-1,3-cyclohexanedione **3** in refluxing tetrahydrofuran (THF) and triethylamine (NEt_3_) to give crude trione **4** that was converted into (±)-**1** in toluene and pyrrolidine at 110 °C [6,99].

Compound (+)-**1** was obtained instead by the intramolecular aldol reaction of **4** in dimethylsulfoxide (DMSO) at 90 °C in the presence of l-phenylalanine and 1 M HClO_4_. The enantiomeric excess (ee) by which (+)-**1** was obtained increased from 87% to 96% after three crystallizations from *n*-hexane. The ee was determined by high performance liquid chromatography (HPLC) equipped with a chiral stationary phase column [99,105].

**Scheme 4:**
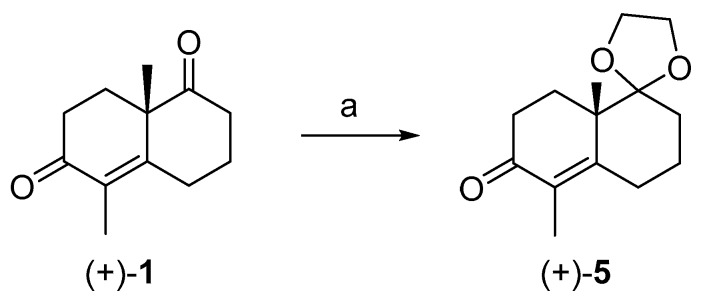
Preparation of (+)-**5**. Reaction conditions: a—1,2-ethanediol, TsOH, molecular sieves, room temperature (rt), 80% yield.

Compound **5** was also prepared both in racemic and in the enantiomerically pure *S* form. Acetalization of enediones (±)-**1** and (+)-**1** was carried out according the procedure described by Ciceri and Demnitz (A in Scheme 2) [125]. This procedure was chosen to verify whether milder conditions could also cause racemization. Thus, (±)-**1** and (+)-**1** were treated at room temperature (rt) with 1,2-ethanediol and TsOH in the presence of molecular sieves to give (±)-**5** (Scheme 2) and (+)-**5**, respectively (Scheme 4). The ee, with which (+)-**5** was obtained, resulted in the same as that of (+)-**1**. Therefore, no racemization occurred.

The results obtained when the acetalization of (+)-**1** was performed with 1,2-ethanediol and TsOH in the presence of a Dean–Stark apparatus are reported in Table 1. In all cases 1,2-ethanediol and TsOH, dissolved in benzene, were dried by refluxing the mixture in the presence of the Dean–Stark apparatus, for two hours before adding (+)-**1**. The reaction was carried out at different times and different amounts of TsOH. From the analysis of Table 1 (entries 1–3), it can be observed that the (+)-**5** ee decreased with increasing reaction time and that the starting ketone (+)-**1** was almost totally consumed after 5 h. Therefore, the process of racemization certainly depends on the amount of TsOH present. Indeed, when the reaction was carried out with a 1% TsOH/(+)-**1** molar ratio (entry 4) instead of 20% TsOH/(+)-**1** molar ratio (entries 1–3), the acetal ee was the same as that of the starting enedione. It was also noticed that the (+)-**1** ee decreased much slower than that of the acetal (+)-**5** and never reached the same racemization degree. From the latter data, it was hypothesized that racemization might have taken place on the acetal (+)-**5**, which is in equilibrium with the enedione (+)-**1** as long as there is water in the reaction medium.

**Scheme 5:**
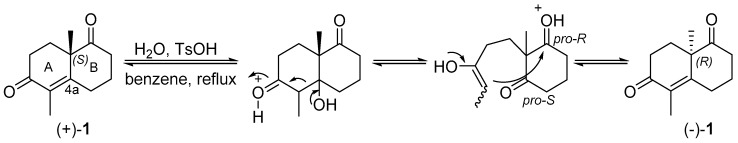
Possible racemization mechanism on *S*-(+)-**1** by the action of water and TsOH in benzene at reflux.

In order to confirm that racemization occurs on the acetal (+)-**5** and not on (+)-**1**, at first, the latter was reacted only with TsOH in benzene at reflux (entry 5) and then with TsOH in benzene with the addition of an increasing amount of H_2_O at reflux (entries 6–8). H_2_O was added to ascertain whether the water formed during the acetalization reaction could have caused the racemization of the enedione (+)-**1**, according to the mechanism described in Scheme 5. 

H_2_O could, indeed, attack the electrophilic C-(4a) in protonated (+)-**1** inducing ring A to open and form a prochiral intermediate, which could cyclize onto *pro-S* C=O, leading to the chiral center racemization. In all of these experiments (entries 5–8) the (+)-**1** ee determined at the end of the reaction was the same as the beginning, even when the reaction was performed in a flask unfitted with a Dean–Stark apparatus (entry 8). Therefore, it appears that this process is not responsible for the racemization.

Once it was established that racemization did not originate from the enedione (+)-**1**, pure acetal (+)-**5** was reacted for 5 h in benzene at reflux with a Dean–Stark apparatus under the reaction conditions reported in Table 2. 

The results obtained when treating (+)-**5** with 1,2-ethanediol in the absence of TsOH showed that the racemization was completely absent under these conditions (entry 1, Table 2); they also showed that racemization was faster (entry 2, Table 2) with respect to that noted starting from (+)-**1** (entry 2, Table 1).

**Scheme 6:**
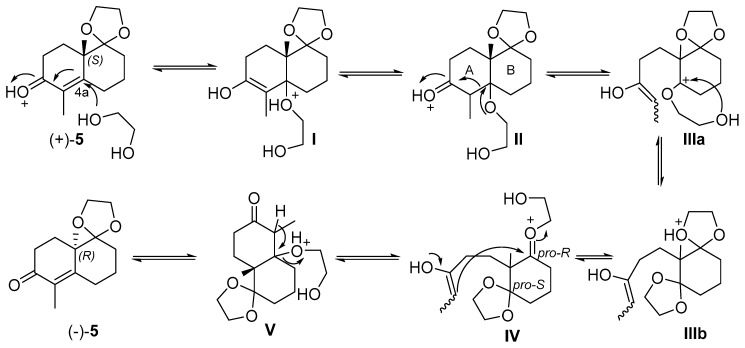
Proposed mechanism for the racemization of (+)-**5** in the course of the acetalization of (+)-**1** with 1,2-ethanediol and TsOH under classical experimental conditions.

Although our intent was not to study the mechanism of that racemization, in agreement with the above data, we hypothesized that it may proceed with the nucleophilic attack of the 1,2-ethanediol on C-(4a) (Scheme 6), leading to the opening of ring A (compound **IIIa** in Scheme 6). Racemization could then occur following the aldol reaction between the enol side chain and the *pro-R* electrophilic C=O on the cycle resulting from the acetal cleavage. 

**Scheme 7:**
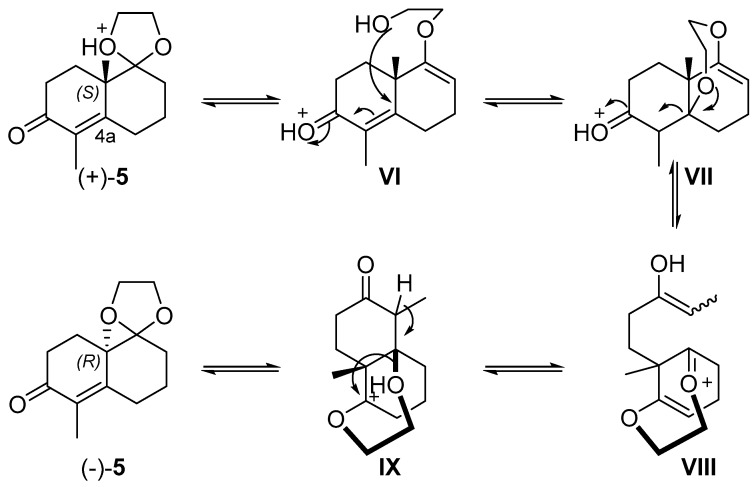
Proposed intramolecular mechanism for the racemization of (+)-**5** in the course of the acetalization of (+)-**1** with 1,2-ethanediol and TsOH under classical experimental conditions.

Acetal (+)-**5** was also treated with TsOH in refluxing benzene in the absence of 1,2-ethanediol for 5 h (entry 3, Table 2). The results obtained in this case showed that almost the same racemization degree was obtained with or without adding 1,2-ethanediol (see also entry 2, Table 1), the only difference between the two cases being that (+)-**1** recovered at the end of the reaction was 2% and 10%, respectively. Such values were in agreement with the greater amount of ethylene glycol present in the second case. According to this last result, it could be possible to imagine an intramolecular racemization mechanism (Scheme 7) that could take place simultaneously with the previous one (Scheme 6). Protonation could cause partial cleavage of the acetal group and the nucleophilic attack on the electrophilic C(4a) of the alcoholic oxygen could cause the opening of the ring (**VIII**, Scheme 7) whereby the subsequent aldol reaction could invert the configuration of the chiral center with the formation of (−)-**5**.

**Scheme 8:**
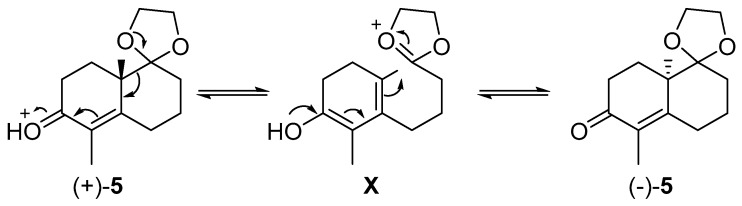
Mechanism proposed by a referee during the article reviewing process.

Furthermore, during the article reviewing process, one of the referees suggested a third possible mechanism that is shown in Scheme 8.

All of the proposed mechanisms (Scheme 6, Scheme 7 and Scheme 8) involve the presence of the α,β-unsaturated system. Therefore, in order to test the validity of such hypothesis, compound (+)-**6** was synthesized starting from (+)-**1** as described in Scheme 8 [126]. Enedione (+)-**1** was chemoselectively deoxygenated at C(6)O through a two-step procedure. The obtaining of compound (+)-**6** was confirmed by nuclear magnetic resonance (NMR) analysis, from which it could be observed the disappearance in the ^13^C-NMR spectrum of the signal of the C(6)O was at 197.2 ppm.

**Scheme 9:**
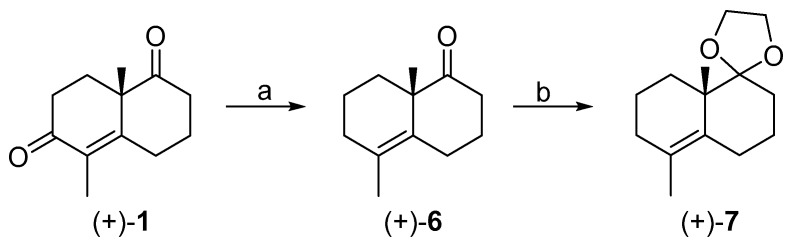
Preparation and acetalization of (+)-**6** under classical experimental conditions. Reaction conditions: a—(i) 1,2-ethanedithiol, TsOH, AcOH, rt; (ii) Raney-Ni, EtOH_abs_, reflux, (21% yield starting from (+)-**1**); b—1,2-ethanediol, TsOH, benzene, reflux, 33% yield.

**Scheme 10:**
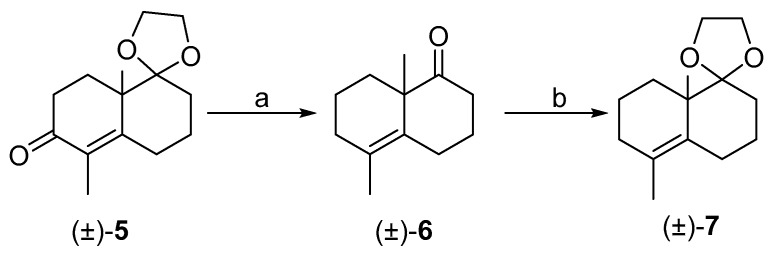
Preparation of (±)-**7**. Reaction conditions: a—(i) 1,2-ethanedithiol, TsOH, AcOH, rt; (ii) Raney-Ni, EtOH_abs_, reflux, (24% yield starting from (±)-**5**); b—1,2-ethanediol, TsOH, molecular sieves, rt, 40% yield.

Compound **6** was synthesized both as the *S* enantiomer (Scheme 9) and as racemate (Scheme 10). In order to have a reference to determine the ee of the compounds obtained during the acetalization reaction of (+)-**6** with 1,2-ethanediol and TsOH, (±)-**6**, obtained from (±)-**5**, was transformed into (±)-**7**. 

The acetalization reaction on ketone (+)-**6** with 1,2-ethanediol and TsOH (20% TsOH/(+)-**6** molar ratio) in refluxing benzene gave (+)-**7** with an ee identical to that of the starting material, demonstrating that the conjugated carbonyl function activates the nucleophilic addition of hydroxy functions which, in turn, are responsible for the racemization.

## 3. Materials and Methods 

All solvents were purchased from Merck Life Sciences S.r.l. (Milano, Italy) and used without further purification unless otherwise noted. Anhydrous tetrahydrofuran (THF) was distilled over sodium/benzophenone. Reactions were monitored by thin layer chromatography (TLC) with precoated silica gel plates with silica gel 60 F254 using UV light as the visualizing agent and phosphomolybdic acid and heat as the developing agent. Column chromatography: silica gel 60, 70–230 mesh ^1^H-NMR spectra were recorded with a Varian Mercury AC 300 at 300.13 MHz or a Bruker AVANCE 400 at 400.13 MHz instruments and ^13^C-NMR spectra were obtained with a Varian Mercury AC 300 at 75.48 MHz or a Bruker AVANCE 400 at 100.61 MHz spectrometers. Chemical shifts were reported as δ values in ppm relative to the residual solvent peak of CDCl_3_ at 7.26 and 77.0 ppm for ^1^H and ^13^C, respectively; J in Hz. Optical rotations were determined for solutions of chloroform (CHCl_3_) with a DIP 370 Jasco digital polarimeter. Analytical high-pressure liquid chromatography (HPLC) was performed with a Shimadzu LC-10AD instrument; RID detector; chiral analytical columns: Phenomenex Lux 3U Amylose-2 4.60 × 50 mm, flow rate 0.5 mL/min for compound **1**, Phenomenex Lux 3U Cellulose-4 4.60 × 50 mm, flow rate 0.5 mL/min for compound **5**, Phenomenex Lux 3U Cellulose-4 4.60 × 150 mm, flow rate 0.8 mL/min for compounds **6** and **7**. GC-MS analysis was carried out on a Shimadzu GCMS-QP5000.

### 3.1. Synthesis of (±)-1

A solution of **3** (0.536 g, 4.25 mmol), **2** (0.540 mL, 5.46 mmol), Et_3_N (0.760 mL, 5.46 mmol), and 5.00 mL of anhydrous THF was stirred under reflux for 2 h. The resulting solution was then cooled to rt, transferred into a one-neck flask using CH_2_Cl_2_, and the excess of ethyl vinyl ketone, Et_3_N and CH_2_Cl_2_ was removed through distillation under reduced pressure. To the resulting mixture, pyrrolidine (0.285 mL, 3.45 mmol) and 25.0 mL of toluene were added under stirring. After the addition, stirring was continued under reflux for 54 h and then cooled to rt. The mixture was then diluted with Et_2_O, washed twice with a 5% HCl solution, with 5% NaOH solution and brine. The organic layers were dried on anhydrous Na_2_SO_4_, filtered, and the solvent evaporated. Purification of the residue on a chromatographic column (CC) (SiO_2_, gradient of EtOAc/*n*-hexane from 15:85 to 40:60) gave enedione **1** (0.238 g, 30% yield) as a yellow oil. The mixture was checked by HPLC analysis on chiral stationary phase column using isopropyl alcohol/*n*-hexane 1:9 as eluent, (+)-**1** t_R_ = 7.1 min, (−)-**1** t_R_ = 8.1 min (Figure S3). ^1^H-NMR (300 MHz, CDCl_3_, Figure S1): δ 1.39 (s, 3H), 1.78 (s, 3H), 1.65–1.85 (m, 1H), 2.00–2.30 (m, 3H), 2.30–2.60 (m, 4H), 2.65 (ddd, J = 16.1, 10.4, 6.0, 1H), 2.94 (dt, J = 15.9, 5.0, 1H).^13^C-NMR (75 MHz, CDCl_3_, Figure S2): δ 11.0 (C10), 21.3 (C9), 23.1, 27.0, 29.4, 33.1, 37.1 (C2, C3, C4, C7, C8), 50.4 (C8a), 130.4 (C5), 158.0 (C4a), 197.2 (C6), 211.7 (C1). GC-MS m/z (rel int. %): 192 (37) [M^+^], 177 (29), 149 (42), 136 (85), 121 (35), 107 (78), 105 (15), 93 (85), 91 (55), 80 (16), 79 (62), 77 (51), 67 (19), 65 (27), 55 (100), 53 (45), 51 (24).

### 3.2. Synthesis of (+)-1 

A solution of **3** (10.0 g, 79.3 mmol), **2** (10.1 mL, 102 mmol), Et_3_N (14.1 mL, 102 mmol), and 90.0 mL of anhydrous THF was stirred under reflux for 3 h. The resulting solution was then cooled to rt, transferred in a one-neck flask using CH_2_Cl_2_, and the excess of ethyl vinyl ketone, Et_3_N and CH_2_Cl_2_ was removed under distillation at reduced pressure. To the resulting mixture, DMSO (133 mL), 1 M HClO_4_ (40.0 mL), and l-phenylalanine (12.3 g, 74.3 mmol) were added and the solution was stirred at 90 °C for 24 h. The resulting mixture was then cooled to rt and poured into cold NaHCO_3_ saturated solution (s.s.) and extracted with EtOAc, the organic layers were then rinsed with brine, dried on anhydrous Na_2_SO_4_, and filtered. After the evaporation of the solvent, the mixture was purified by CC (SiO_2_, gradient of EtOAc/*n*-hexane from 15:85 to 40:60), giving the enedione (+)-**1** (8.67 g, 60% yield) with 87% ee obtained by HPLC analysis on chiral stationary phase column. The ee was enriched through several crystallizations: (+)-**1** was dissolved in *n*-hexane and recrystallized at −20 °C overnight, the supernatant was removed, and the crystals were washed with *n*-hexane and re-subjected to the same crystallization conditions until they reached 96% ee, measured by HPLC analysis on chiral stationary phase column using isopropyl alcohol/*n*-hexane 1:9 as the eluent (Figure S4). 

### 3.3. Synthesis of (±)-5 in Refluxing Benzene

1,2-ethanediol (0.0770 mL, 1.37 mmol) and TsOH (0.0437 g, 0.230 mmol) were dissolved in benzene (20.0 mL) and the solution was stirred under reflux using a Dean–Stark apparatus to remove H_2_O from the reagents. After 1.5 h, the enedione (±)-**1** (0.220 g, 1.14 mmol) was added and the stirring was continued under reflux for 2.5 h. After cooling to rt, the solution was diluted with EtOAc, poured into NaHCO_3_ s.s., and the two phases were separated. The organic layers were rinsed with brine, dried over anhydrous Na_2_SO_4_, and filtered. Evaporation of solvent followed by CC (SiO_2_, gradient of EtOAc/*n*-hexane from 15:85 to 40:60) of the residue afforded the acetal (±)-**5** (0.189 g, 70% yield) as a pale-yellow oil. The mixture was checked by HPLC analysis on a chiral stationary phase column using isopropyl alcohol/*n*-hexane 1:9 as the eluent, (−)-**5** t_R_ = 6.0 min, (+)-**5** t_R_ = 7.4 min (Figure S7). ^1^H-NMR (300 MHz, CDCl_3_, Figure S5): δ 1.31 (s, 3H), 1.75 (s, 3H), 1.50–1.96 (m, 5H), 2.06–2.40 (m, 4H), 2.60-2.80 (m, 1H), 3.80-4.05 (m, 4H). ^13^C-NMR (75 MHz, CDCl_3_, Figure S6): δ 11.3 (C10), 20.7 (C9), 21.3, 26.3, 26.4, 29.6, 33.5 (C2, C3, C4, C7, C8), 45.1 (C8a), 64.9, 65.2 (C11, C12), 112.6 (C1), 129.9 (C5), 160.0 (C4a), 198.5 (C6). GC-MS *m/z* (rel int. %) 236 (3) [M^+^], 100 (5), 99 (100), 55 (19).

### 3.4. Synthesis of (+)-5 at Rt

Enedione (+)-**1** (1.00 g, 5.20 mmol) was dissolved in 1,2-ethanediol (28.0 mL) containing molecular sieves. TsOH monohydrate (0.989 g, 5.20 mmol) was added all at once and the solution was stirred at rt for 40 min under an Ar atmosphere. The solution was then diluted with EtOAc, poured into NaHCO_3_ s.s., and the two phases were separated. The organic layers were then rinsed with brine, dried over anhydrous Na_2_SO_4_, and filtered. After the evaporation of the solvent, the mixture was purified by CC (SiO_2_, gradient of EtOAc/*n*-hexane from 15:85 to 40:60) to give acetal (+)-**5** (0.983 g, 80% yield) as a pale yellow oil with 96% ee, as measured by HPLC analysis on a chiral stationary phase column using isopropyl alcohol/*n*-hexane 1:9 as the eluent (Figure S8).

### 3.5. Acetalization of (+)-1 under Classical Condition in Refluxing Benzene

The reaction was performed as described for the preparation of (±)-**5** starting from (+)-**1** (0.192 g, 1 mmol, 96% ee) and the mixture was refluxed for the time indicated on Table 1. The ee with which (+)-**5** was obtained is reported in Table 1.

### 3.6. Synthesis of (±)-6

To a solution of (±)-**5** (0.752 g, 3.20 mmol) in glacial AcOH (1.52 mL), 1,2-ethanedithiol (0.294 mL, 3.52 mmol), TsOH (0.285 g, 1.50 mmol), and glacial AcOH (3.42 mL) were added under an Ar atmosphere. The mixture was stirred for 4.5 h at rt and then poured into water and stirred for another 15 min. The residue was diluted with CH_2_Cl_2_, the two phases were separated, and the organic one washed with water, NaHCO_3_ s.s., dried over anhydrous Na_2_SO_4_, filtered, and the solvent was evaporated. Activated Raney-Ni (0.196 g) was added under an Ar atmosphere to the residue dissolved in EtOH_abs_ (10.0 mL) and the solution was refluxed for 3 h. After cooling to rt, the solution was filtered off to remove Raney-Ni, washed with EtOH, with CH_2_Cl_2_ and the organic solvents were distilled off through a Vigreux column. The residue was purified by CC (SiO_2_, gradient of Et_2_O/*n*-hexane from 3:97 to 10:90), to give (±)-**6** (0.0412 g, 24% yield) as a volatile colorless oil. The racemic mixture was analyzed by HPLC on a chiral stationary phase column using isopropyl alcohol/*n*-hexane 1:99 as the eluent, (−)-**6** t_R_ = 5.1 min, (+)-**6** t_R_ = 5.5 min (Figure S11). ^1^H-NMR (400 MHz, CDCl_3_, Figure S9): δ 1.26 (s, 3H), 1.65 (s, 3H), 1.48–1.72 (m, 4H), 1.84–2.08 (m, 4H), 2.15–2.27 (m, 1H), 2.26–2.36 (m, 1H), 2.55–2.64 (m, 1H), 2.65–2.72 (m, 1H). ^13^C-NMR (100 MHz, CDCl_3_, Figure S10): δ 19.0, 19.8, 24.4, 24.6, 25.6, 31.7, 32.5 (C3, C4, C6, C7, C8, C9, C10), 38.1 (C2), 50.7 (C8a), 128.5, 132.2 (C4a, C5), 215.6 (C1). HRMS: calcd. for C_12_H_18_O [M+Na]^+^: 201.1255; found 201.1249. GC-MS *m/z* (rel int. %) 178 (19) [M^+^], 163 (9), 136 (12), 135 (100), 107 (29), 105 (7), 94 (5), 93 (32), 91 (22), 81 (8), 79 (31), 77 (16), 67 (14), 65 (9), 55 (14), 53 (10), 51 (6).

### 3.7. Synthesis of (+)-6

To a solution of (+)-**1** (1.31 g, 6.82 mmol) in glacial AcOH (3.00 mL) were added 1,2-ethanedithiol (0.630 mL, 7.51 mmol), TsOH (0.611 g, 3.21 mmol), and glacial AcOH (8.36 mL) under an Ar atmosphere. The mixture was stirred for 4.5 h at rt and after that period poured into water and stirred for another 20 min. The white solid was filtered off through a Celite pad, washed with water, NaHCO_3_ s.s., washed again with water, and dried. The aqueous layers were extracted with CH_2_Cl_2_ that were dried over anhydrous Na_2_SO_4_, filtered, and dried. The collected residues were treated under an Ar atmosphere with activated Raney-Ni (1.00 g) in EtOH_abs_ (50.0 mL) and the solution was refluxed for 3 h. After cooling to rt, the solution was filtered off to remove Raney-Ni, washed with EtOH, with CH_2_Cl_2_, and the organic solvents were distilled off through a Vigreux column. The residue was purified by CC (SiO_2_, gradient of Et_2_O/*n*-hexane from 3:97 to 10:90) to give (+)-**6** (0.0412 g, 24% yield) as a volatile colorless oil. The optical purity was measured by HPLC analysis on a chiral stationary phase column using isopropyl alcohol/*n*-hexane 1:99 as the eluent, confirming 96% ee (Figure S12). [α]_D_^25^ (c = 46.2 mg/mL in CHCl_3_) = +127.4. 

### 3.8. Synthesis of (±)-7

Ketone (±)-**6** (0.0400 g, 0.224 mmol) was dissolved in 1,2-ethanediol (1.13 mL) containing molecular sieves. TsOH monohydrate (0.0427 g, 0.224 mmol) was added all at once and the solution was stirred at rt for 40 min. The solution was poured into NaHCO_3_ s.s. and Et_2_O was added. The two phases were separated and the organic one was then rinsed with brine, dried over anhydrous Na_2_SO_4_, filtered, and evaporated to dryness. The residue was purified by CC (SiO_2_, gradient of CH_2_Cl_2_/*n*-hexane from 20:80 to 40:60) to give acetal (±)-**7** (0.0202 g, 40% yield) as a pale-yellow oil. The racemic mixture was analyzed by HPLC analysis on a chiral stationary phase column using *n*-hexane as eluent, (+)-**7** t_R_ = 3.9 min, (−)-**7** t_R_ = 4.9 min (Figure S15). ^1^H-NMR (400 MHz, C_6_D_6_, Figure S13): δ 1.36 (s, 3H), 1.60 (s, 3H), 1.47-1.76 (m, 6H), 1.77–1.95 (m, 3H), 1.99–2.11 (m, 2H), 2.49–2.57 (m, 1H), 3.43–3.63 (m, 4H). ^13^C-NMR (100 MHz, C_6_D_6_, Figure S14): δ 19.8, 20.2, 23.4, 23.5, 24.8, 30.1, 31.2, 33.2 (C2, C3, C4, C6, C7, C8, C9, C10), 44.7 (C8a), 64.9, 65.2 (C11, C12), 113.8 (C1), 126.4, 133.8 (C4a, C5). HRMS: calcd. for C_14_H_22_O_2_ [M+Na]^+^: 245.1517; found 245.1510. GC-MS *m/z* (rel int. %) 222 (3) [M^+^], 100 (6), 99 (100), 55 (14).

### 3.9. Synthesis of (+)-7

1,2-ethanediol (0.0187 mL, 0.336 mmol) and TsOH (0.0106 g, 0.0560 mmol) were dissolved in benzene (5.00 mL) and the solution was stirred under reflux using a Dean–Stark apparatus to remove H_2_O from the reagents. After 1.5 h, the ketone (+)-**6** (0.0536 g, 0.280 mmol) was added to the solution and stirring was continued under reflux for another 2.5 h. After cooling to rt, the solution was diluted with EtOAc, poured into NaHCO_3_ s.s., and the two phases were separated. The organic layers were rinsed with brine, dried over anhydrous Na_2_SO_4_, and filtered. Evaporation of the solvent followed by CC (SiO_2_, gradient of CH_2_Cl_2_/*n*-hexane from 20:80 to 40:60) of the residue afforded the acetal (+)-**7** (0.025 g, 33% yield) as a pale-yellow oil. The optical purity was measured by HPLC analysis on a chiral stationary phase column using *n*-hexane as the eluent confirming 96% ee (Figure S16). [α]_D_^25^ (c = 9.73 mg/mL in CHCl_3_) = + 84.4.

## 4. Conclusions

In this work, we have shown that the acetalization of enedione (+)-**1** with TsOH and 1,2-ethanediol in refluxing benzene caused the racemization of the corresponding acetal (+)-**5** unless conditions reported in Table 1, entry 4 are adopted. Mechanism hypotheses for the racemization suggest the nucleophilic attack of an alcoholic oxygen, intermolecular (1,2-ethanediol), or intramolecular on the electrophilic β carbon of the α,β-unsaturated system and consequently ring A opening with loss of the chiral information. The re-evaluation of the optical purity of the acetalized material (+)-**5** or (−)-**5**, when this classical procedure is adopted by chiral HPLC, seems anyway to be a due step. 

## Figures and Tables

**Table 1:** Acetalization of (+)-**1** under the classical condition ^1^.

Entry	Reaction Time (h)	TsOH (mmol)	1,2-Ethanediol (mmol)	(+)-5 ee (%) ^2^	(+)-1 ee (%) ^2^	(+)-1 (%) ^3^	H_2_O (mmol) ^4^
1	2.5	0.2	1.2	60	80	7	-
2	5	0.2	1.2	30	66	2	-
3	24	0.2	1.2	10	60	1	-
4	2.5	0.01	14.5	96	96	4	-
5	2.5	0.2	-	-	96	100	-
6	2.5	0.2	-	-	96	100	1
7	2.5	0.2	-	-	96	100	53
8	2.5	0.2	-	-	96	100	53 ^5^

^1^ Reaction conditions: (+)-**1** (1 mmol, 96% ee), benzene (20 mL), 1,2-ethanediol, TsOH, Dean–Stark apparatus, reflux, 70% yield. ^2^ The ee was determined by high performance liquid chromatography (HPLC) equipped with a chiral stationary phase column. ^3^ Percentage of (+)-**1** recovered after quenching the reaction. ^4^ H_2_O added in the reaction flask. ^5^ The reaction was performed in a flask unfitted with a Dean–Stark apparatus.

**Table 2:** Reaction of (+)-**5** ^1^ with ethanediol.

Entry	TsOH (mmol)	1,2-Ethanediol (mmol)	(+)-5 ee (%) ^2^	(+)-1 ee (%) ^2^	(+)-1 (%) ^3^
1	-	1.2	96	-	0
2	0.2	1.2	12	-	0
3	0.2	-	34	70	10

^1^ Reaction conditions: (+)-**5** (1 mmol, 96% ee), benzene (20 mL), Dean–Stark apparatus, reflux, 5 h. ^2^ The ee was determined by HPLC equipped with a chiral stationary phase column. ^3^ Percentage of (+)-**1** recovered after quenching the reaction.

## Supplementary Materials

Supplementary materials can be found at https://www.mdpi.com/1422-0067/20/24/6147/s1. ^1^H, ^13^C-NMR spectra and HPLC chromatograms of all synthesized compounds.
