# Supplementary material for: Unexpected Racemization in the Course of the Acetalization of (+)-(S)-5-Methyl-Wieland–Miescher Ketone with 1,2-Ethanediol and TsOH under Classical Experimental Conditions
Source: Int J Mol Sci. 2019 Dec 5;20(24):6147. doi: 10.3390/ijms20246147 (PMC6941009; doi:10.3390/ijms20246147)
Supplement: Supplementary file 1 [file ijms-20-06147-s001.pdf]

Supporting Information

Pag 2 ^{1}H-NMR (300 MHz, $CDCl_3$) of compound (±)-**1**

Pag 3 ^{13}C-NMR (75 MHz, $CDCl_3$) of compound (±)-**1**

Pag 4 HPLC chromatogram (Phenomenex, Lux 3U Amylose-2, 4.60 x 50 mm) of compound (±)-**1**

Pag 5 HPLC chromatogram (Phenomenex, Lux 3U Amylose-2, 4.60 x 50 mm) of compound (+)-**1**

Pag 6 ^{1}H-NMR (300 MHz, $CDCl_3$) of compound (±)-**5**

Pag 7 ^{13}C-NMR (75 MHz, $CDCl_3$) of compound (±)-**5**

Pag 8 HPLC chromatogram (Phenomenex Lux 3U Cellulose-4 4.60 x 50 mm) of compound (±)-**5**

Pag 9 HPLC chromatogram (Phenomenex Lux 3U Cellulose-4 4.60 x 50 mm) of compound (+)-**5**

Pag 10 ^{1}H-NMR (400 MHz, $CDCl_3$) of compound (±)-**6**

Pag 11 ^{13}C-NMR (100 MHz, $CDCl_3$) of compound (±)-**6**

Pag 12 HPLC chromatogram (Phenomenex Lux 3U Cellulose-4 4.60 x 150 mm) of compound (±)-**6**

Pag 13 HPLC chromatogram (Phenomenex Lux 3U Cellulose-4 4.60 x 150 mm) of compound (±)-**6**

Pag 14 ^{1}H-NMR (400 MHz, C_6D_6) of compound (±)-**7**

Pag 15 ^{13}C-NMR (100 MHz, C_6D_6) of compound (±)-**7**

Pag 16 HPLC chromatogram (Phenomenex Lux 3U Cellulose-4 4.60 x 150 mm) of compound (±)-**7**

Pag 17 HPLC chromatogram (Phenomenex Lux 3U Cellulose-4 4.60 x 150 mm) of compound (+)-**7**

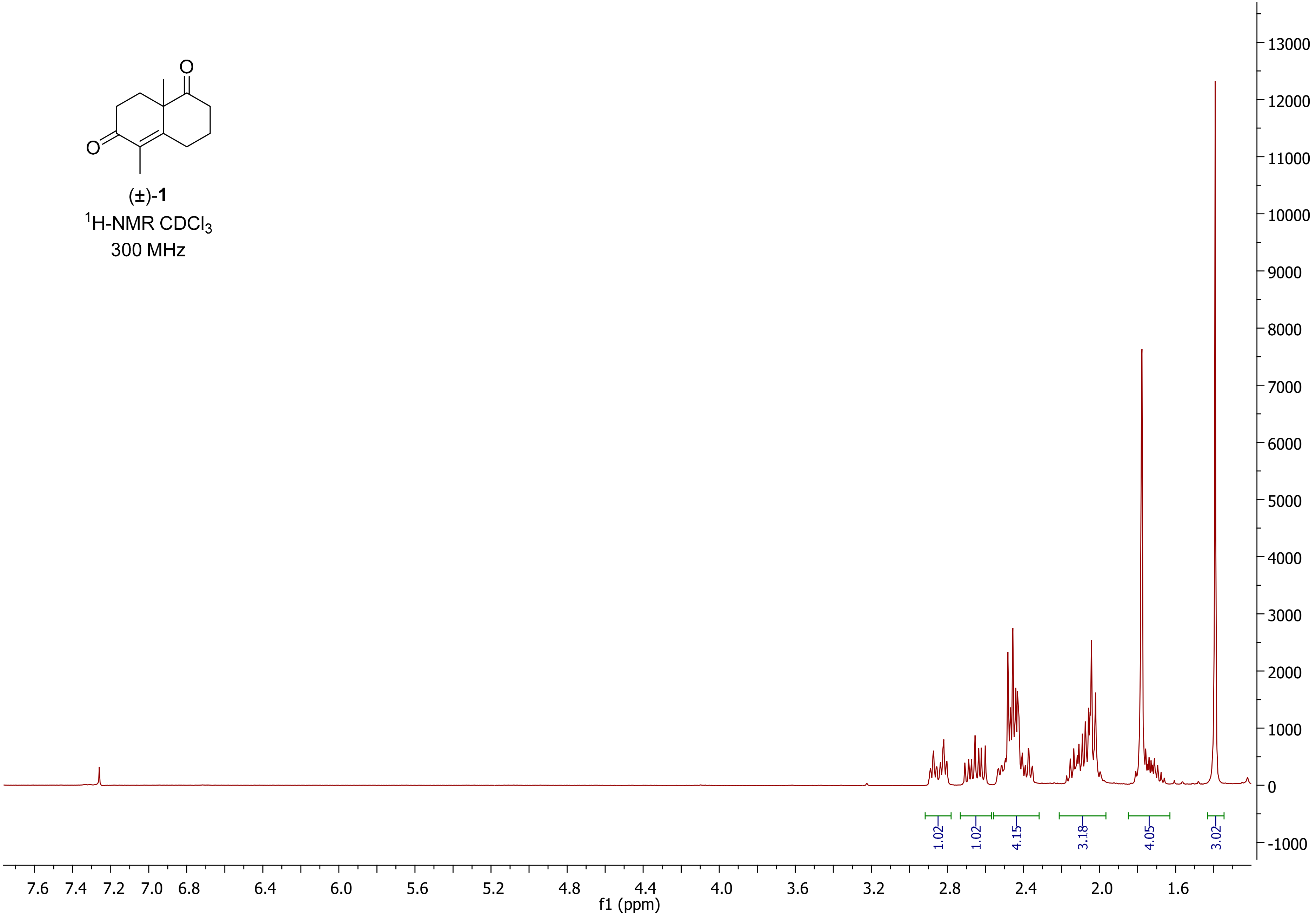

O
O
(±)-1
1H-NMR CDCl3
300 MHz
1.02
1.02
4.15
3.18
4.05
3.02
13000
12000
11000
10000
9000
8000
7000
6000
5000
4000
3000
2000
1000
0
-1000
7.6 7.4 7.2 7.0 6.8 6.4 6.0 5.6 5.2 4.8 4.4 4.0 3.6 3.2 2.8 2.4 2.0 1.6
f1 (ppm)

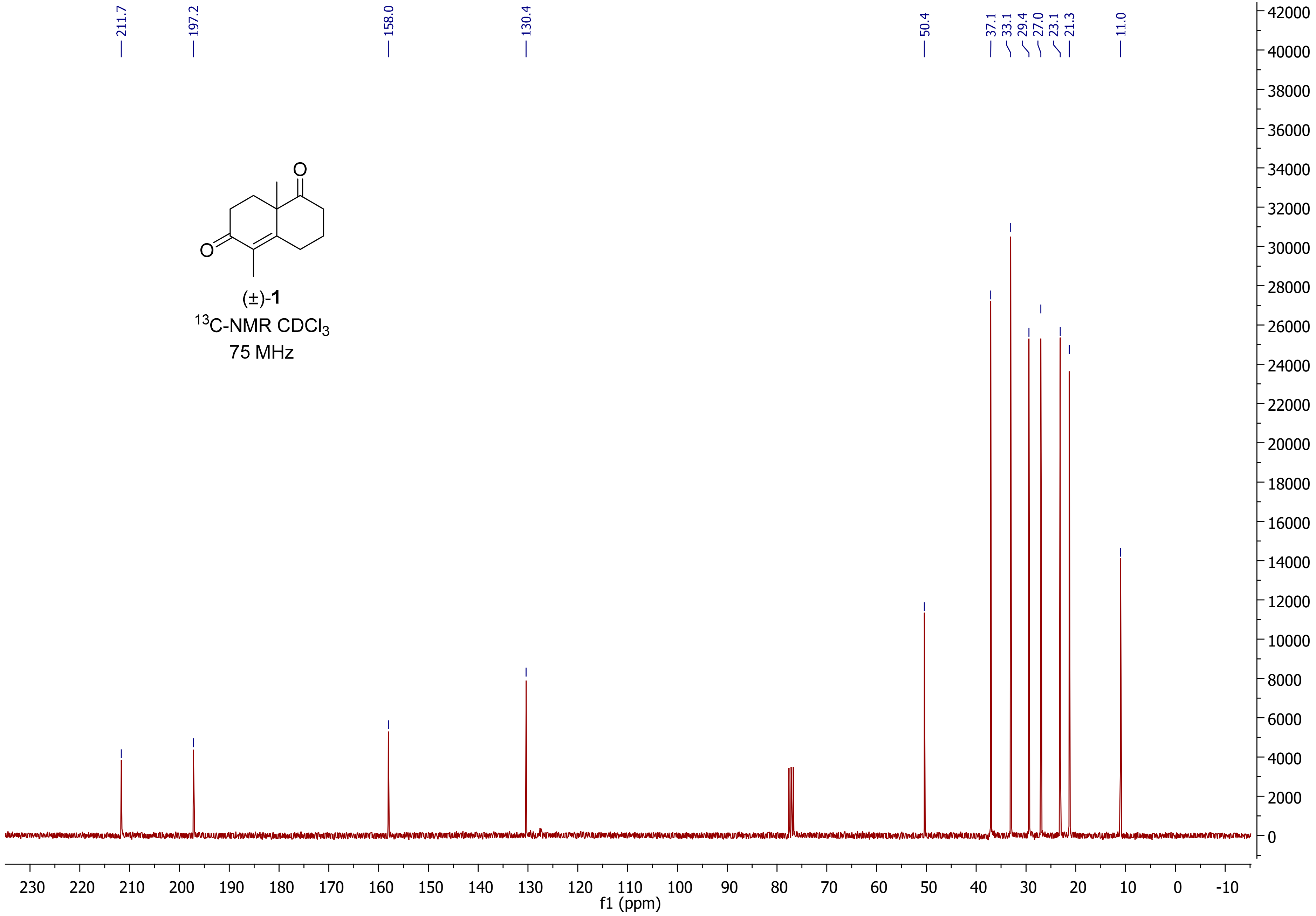
211.7
197.2
158.0
130.4
50.4
37.1
33.1
29.4
27.0
23.1
21.3
11.0
O
O
(±)-**1**
13C-NMR CDCl3
75 MHz
42000
40000
38000
36000
34000
32000
30000
28000
26000
24000
22000
20000
18000
16000
14000
12000
10000
8000
6000
4000
2000
0
230 220 210 200 190 180 170 160 150 140 130 120 110 100 90 80 70 60 50 40 30 20 10 0 -10
f1 (ppm)

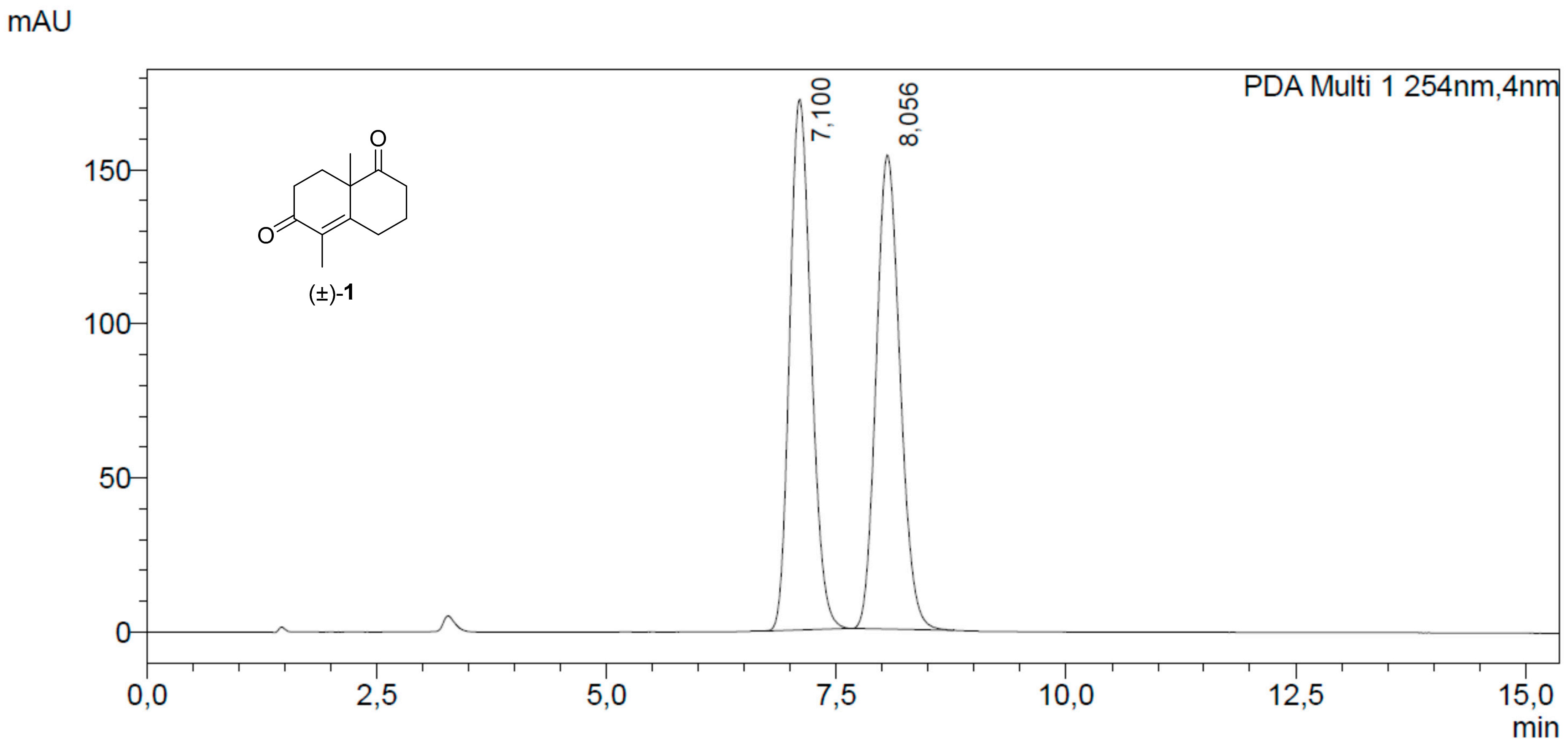

Column: Phenomenex, Lux 3U Amylose-2, 4.60 x 50 mm;

Flow rate: 0.5 mL/min

Eluent: isopropyl alcohol/*n*-hexane 1:9

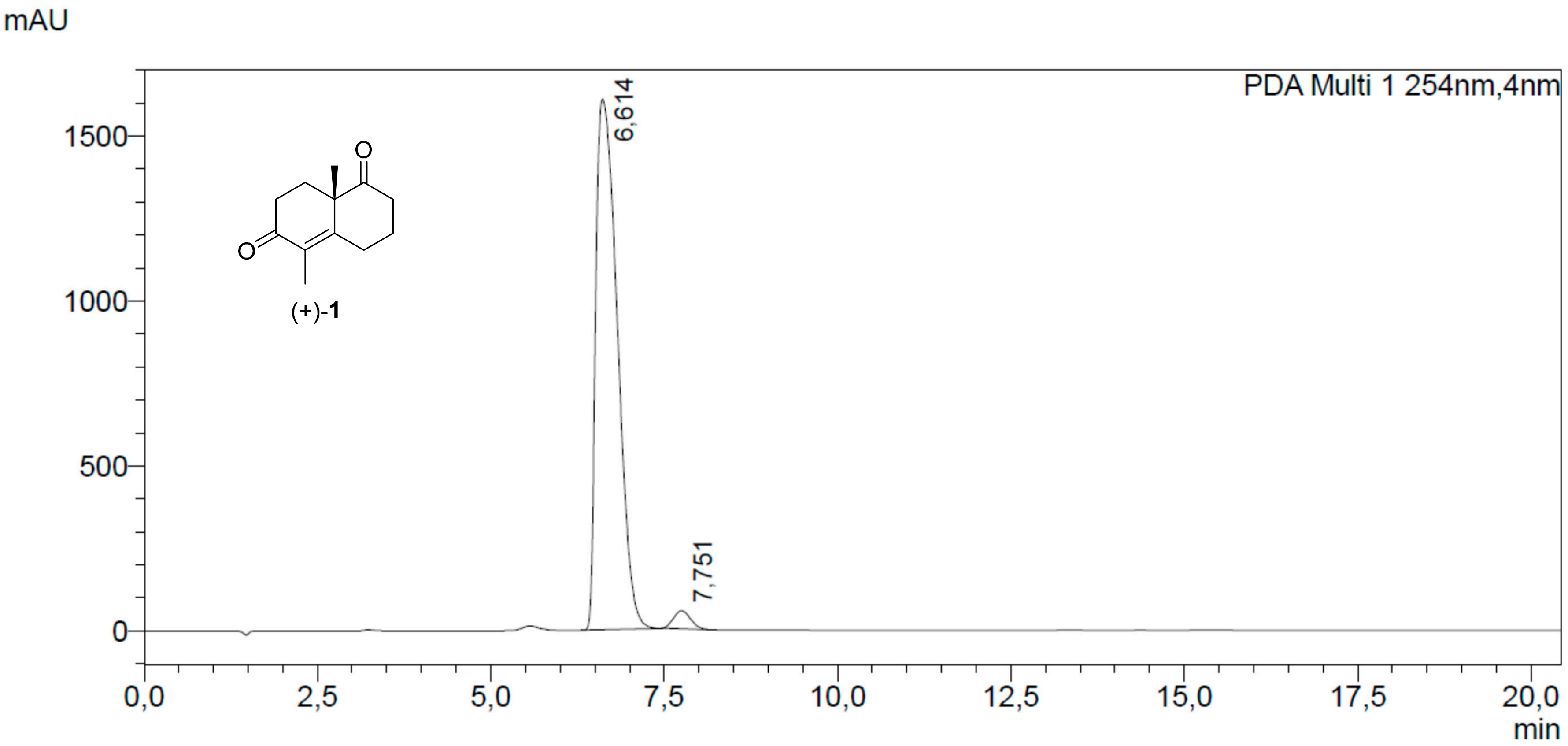

Column: Phenomenex, Lux 3U Amylose-2, 4.60 x 50 mm;

Flow rate: 0.5 mL/min

Eluent: isopropyl alcohol/*n*-hexane 1:9

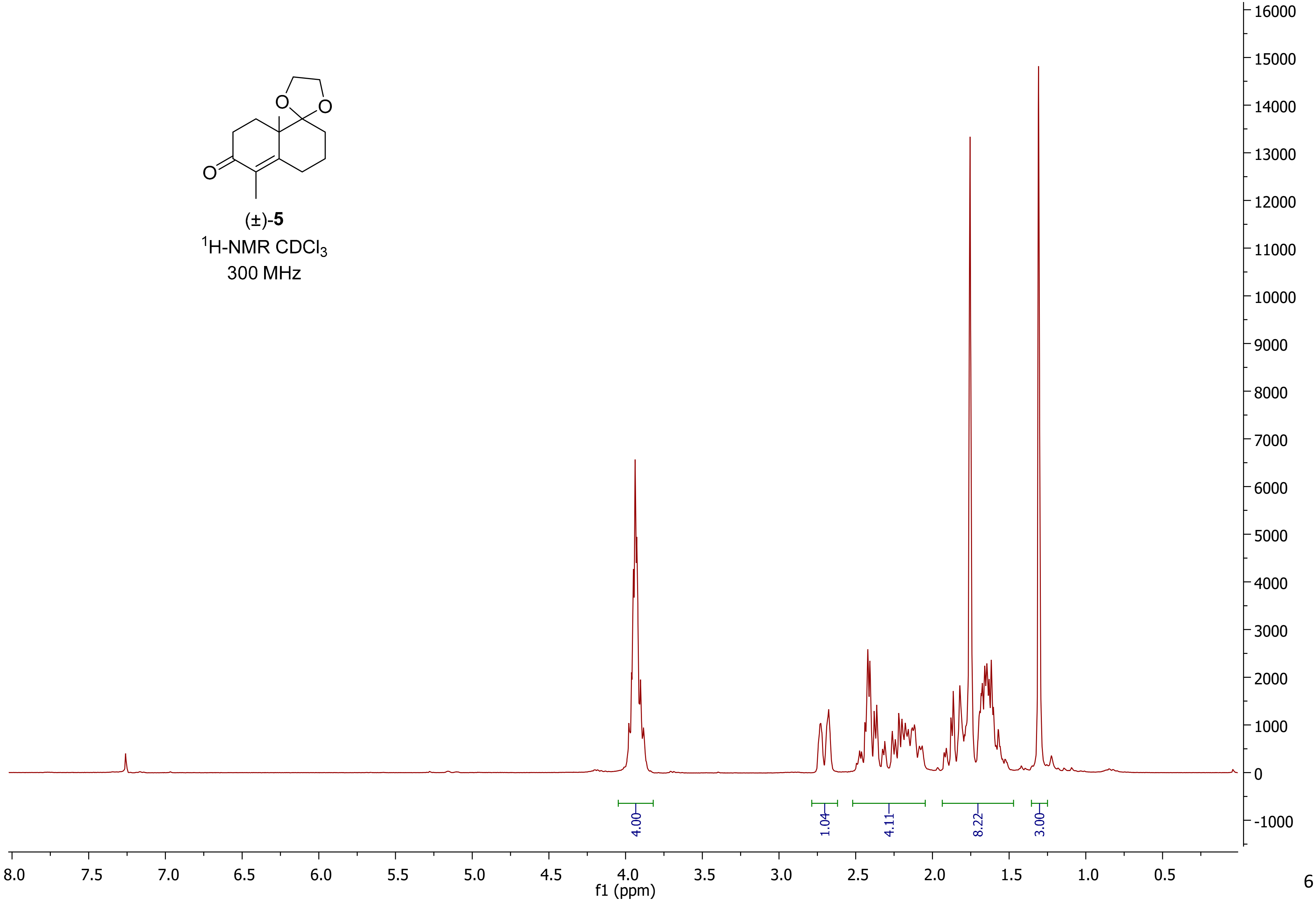
(±)-**5**
^{1}H-NMR $CDCl_3$
300 MHz
4.00
1.04
4.11
8.22
3.00
f1 (ppm)

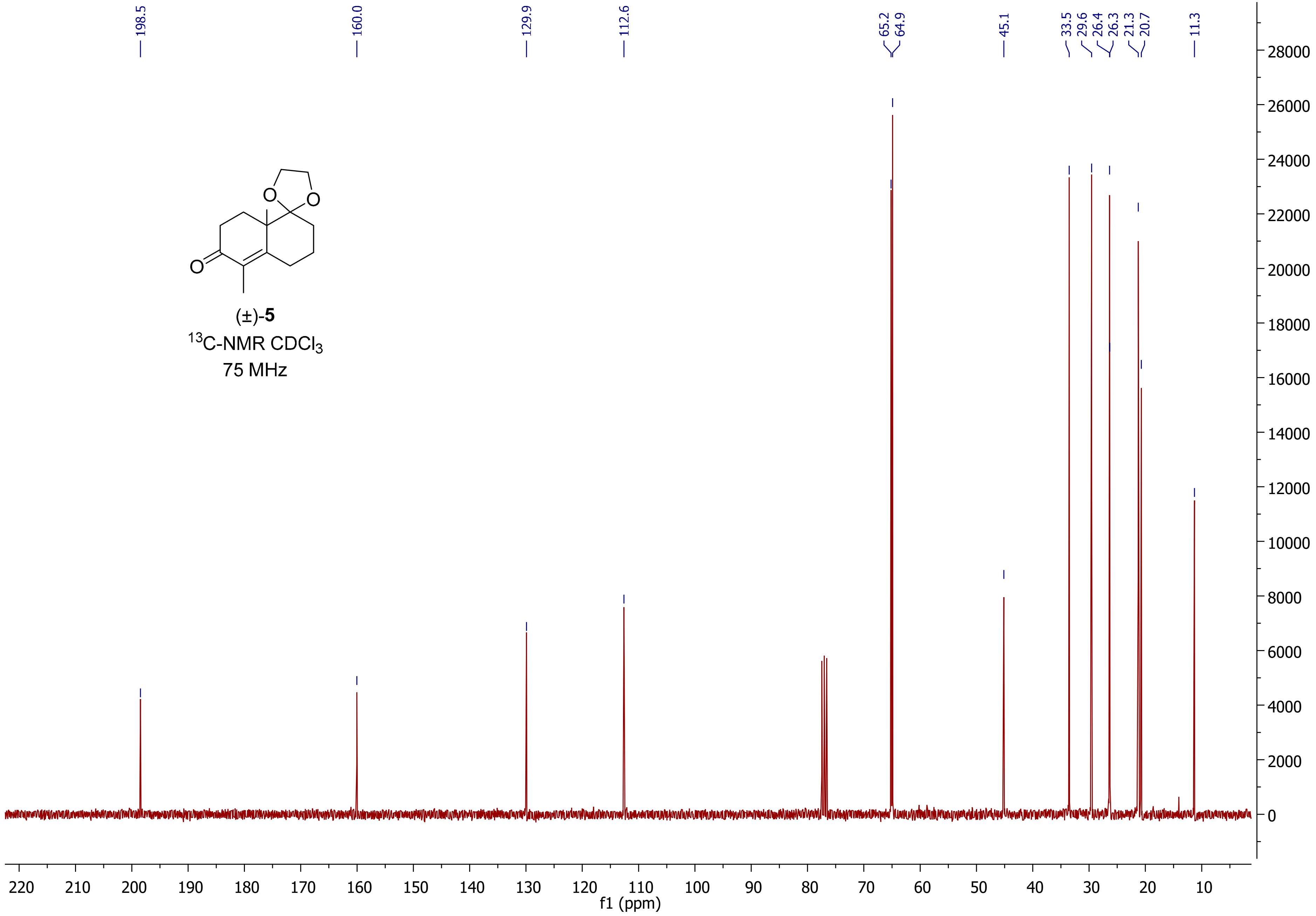
198.5
160.0
129.9
112.6
65.2
64.9
45.1
33.5
29.6
26.4
26.3
21.3
20.7
11.3
(±)-5
13C-NMR CDCl3
75 MHz
f1 (ppm)

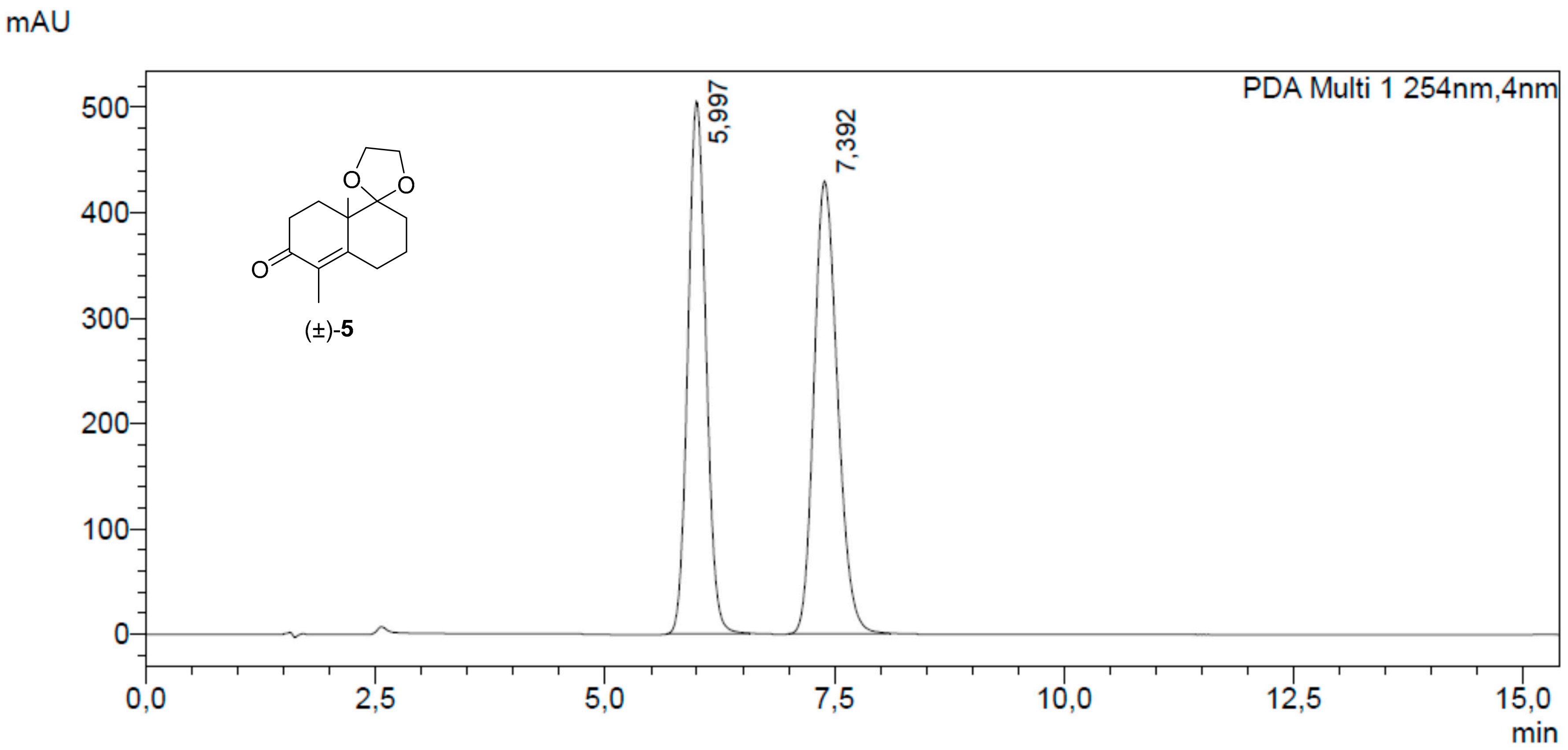

Column: Phenomenex Lux 3U Cellulose-4 4.60 x 50 mm;

Flow rate: 0.5 mL/min

Eluent: isopropyl alcohol/*n*-hexane 1:9

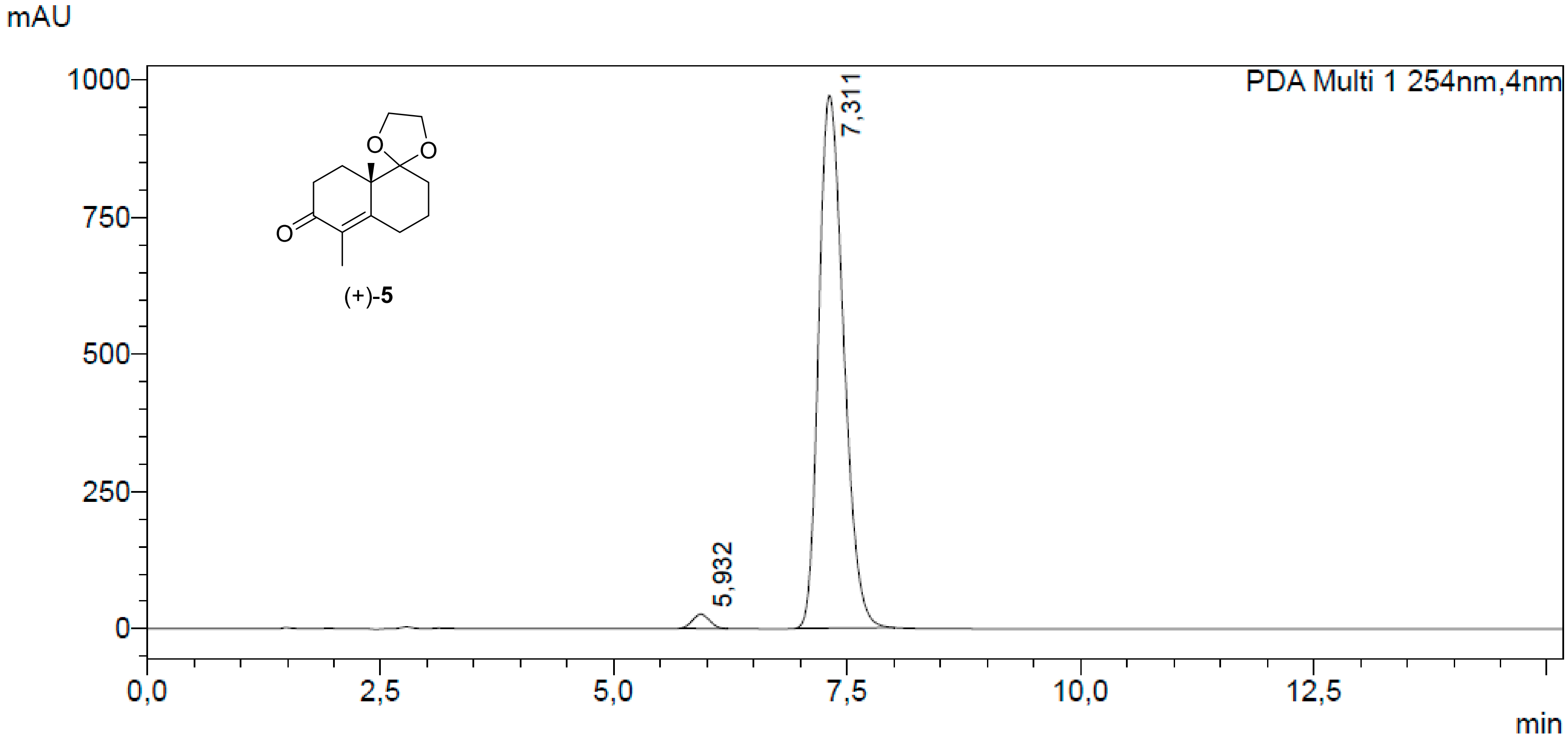

Column: Phenomenex Lux 3U Cellulose-4 4.60 x 50 mm;

Flow rate: 0.5 mL/min

Eluent: isopropyl alcohol/*n*-hexane 1:9

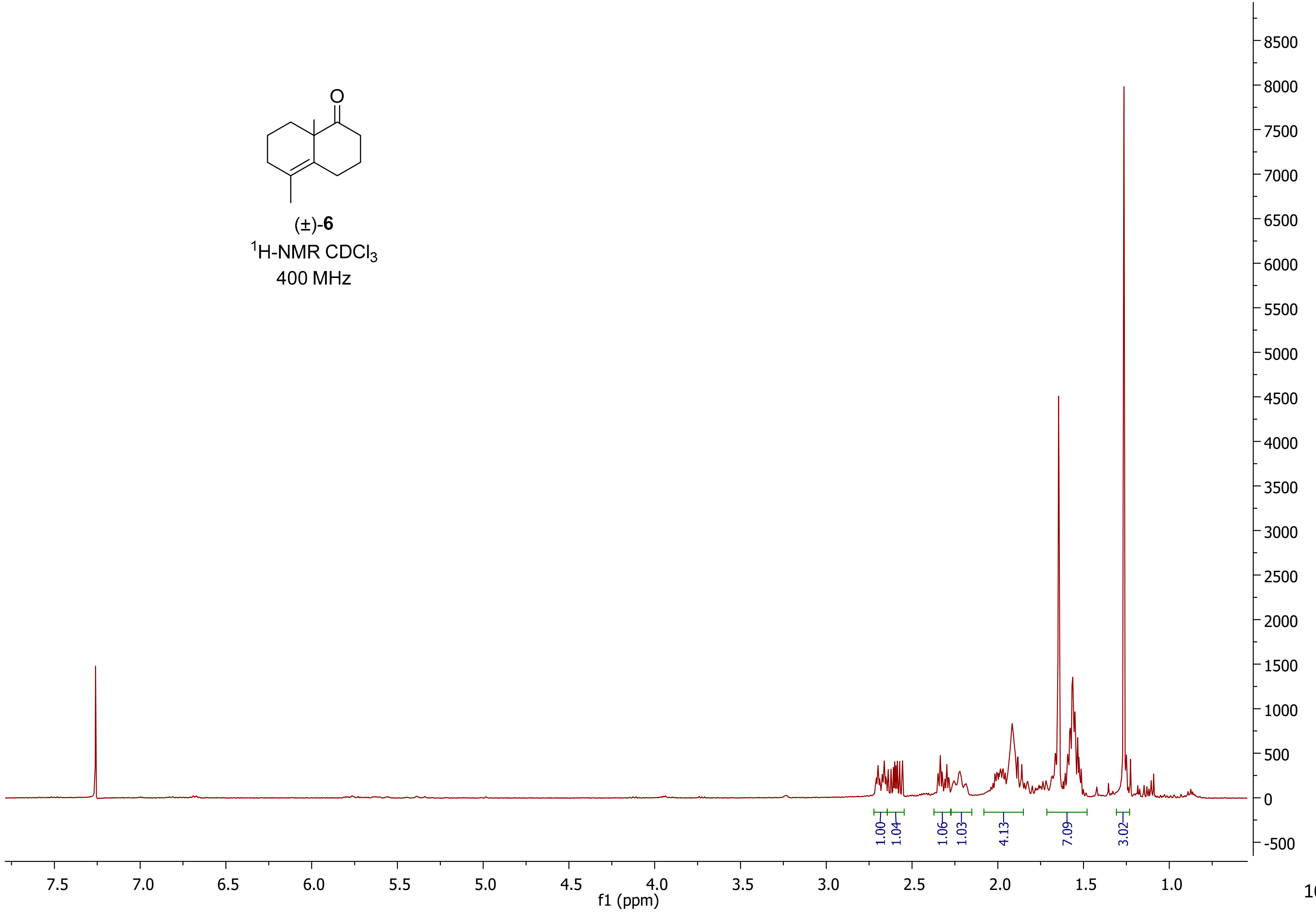

O
(±)-6
1H-NMR CDCl3
400 MHz
1.00
1.04
1.06
1.03
4.13
7.09
3.02
f1 (ppm)

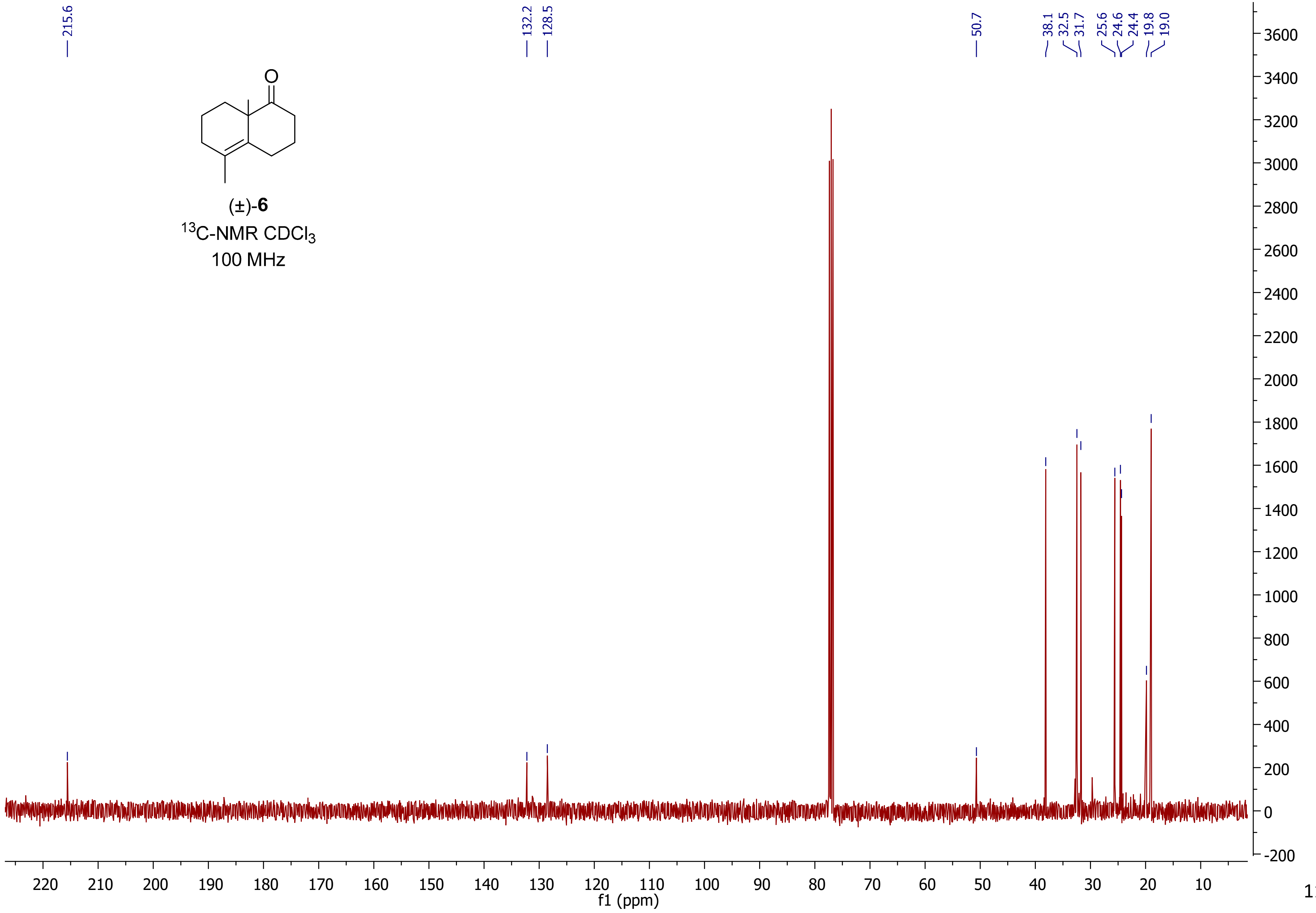

(±)-**6**
^{13}C-NMR CDCl$_3$
100 MHz
215.6
132.2
128.5
50.7
38.1
32.5
31.7
25.6
24.6
24.4
19.8
19.0
f1 (ppm)

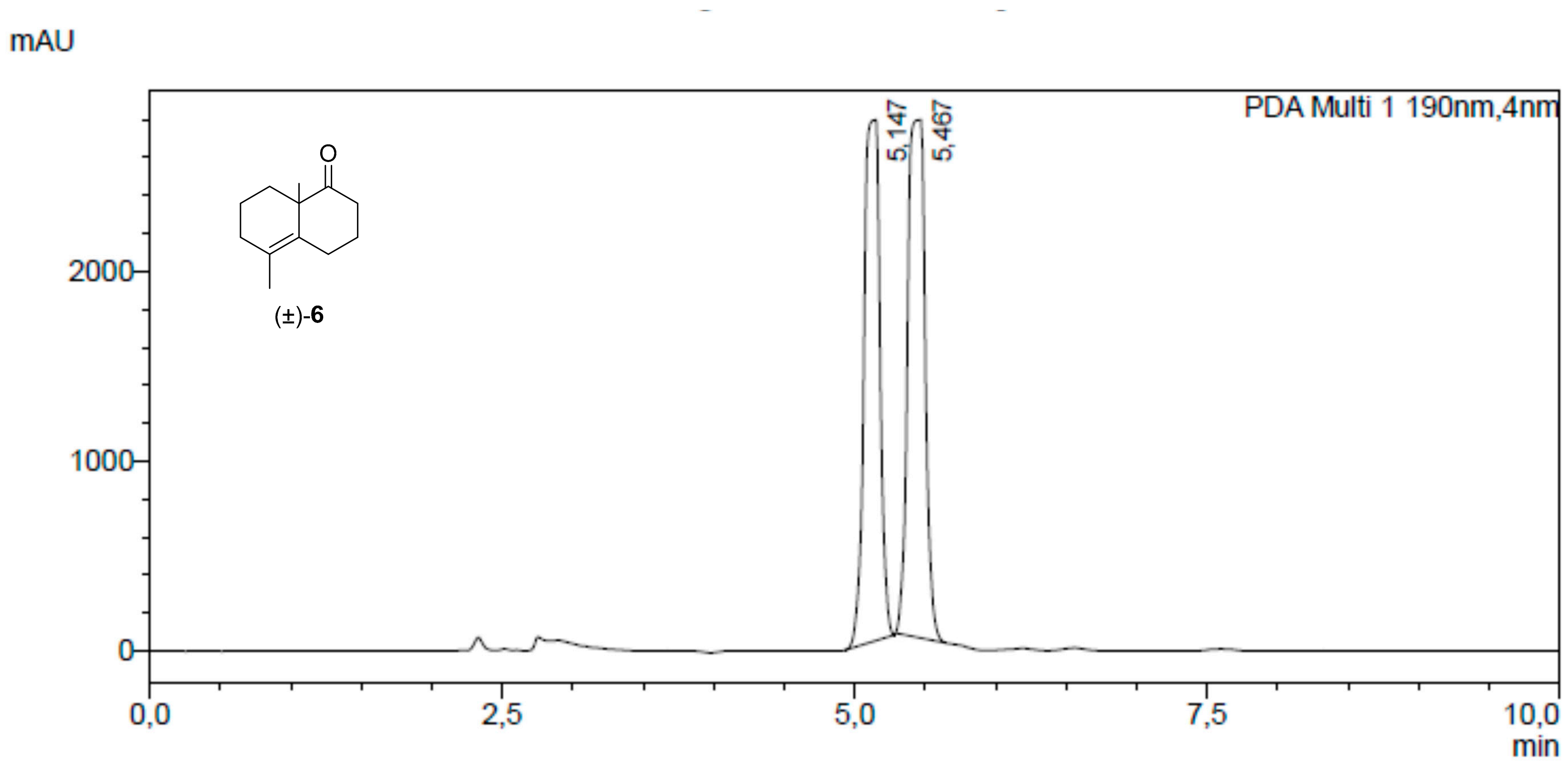

Column: Phenomenex Lux 3U Cellulose-4 4.60 x 150 mm;

Flow rate: 0.8 mL/min

Eluent: isopropyl alcohol/*n*-hexane 1:99

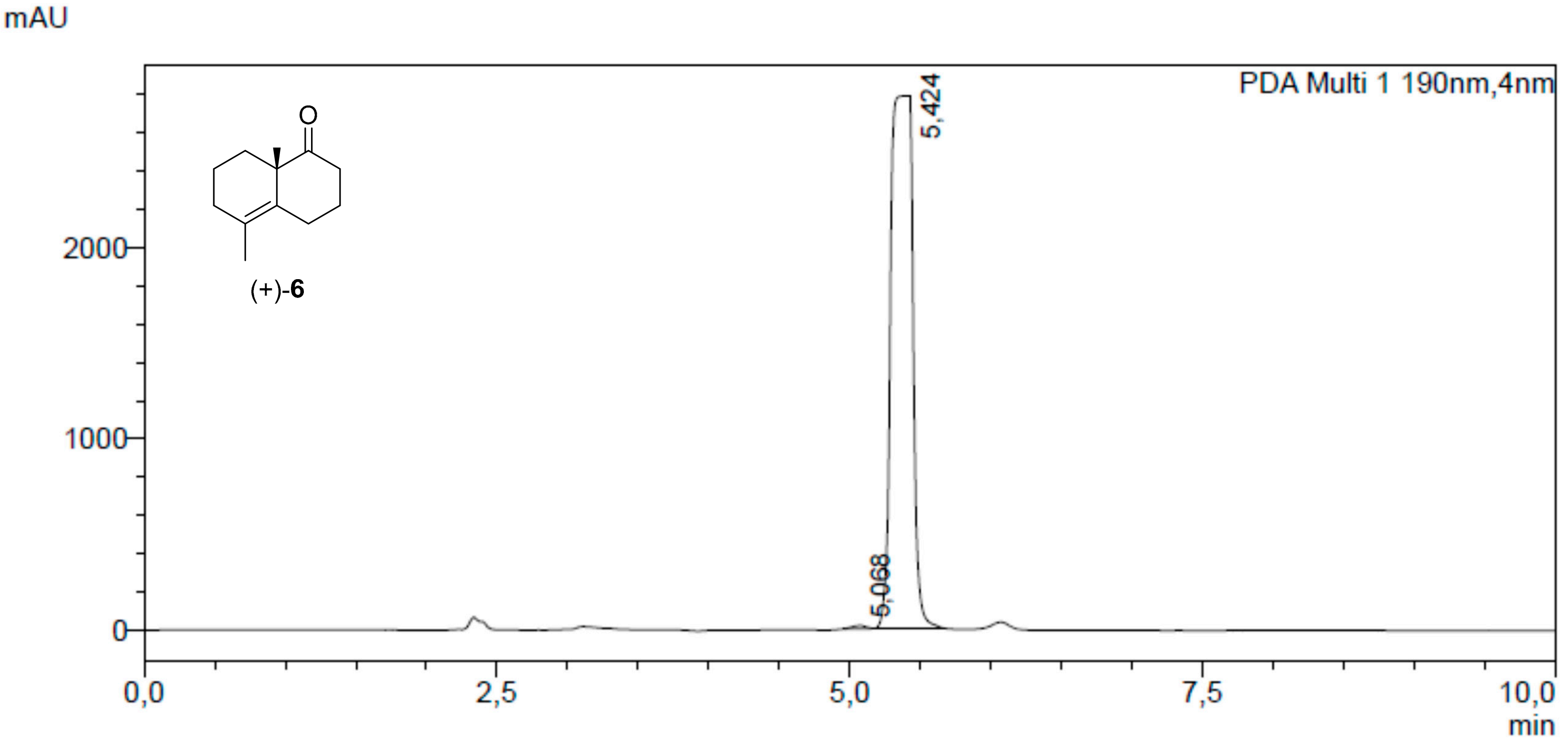

Column: Phenomenex Lux 3U Cellulose-4 4.60 x 150 mm;

Flow rate: 0.8 mL/min

Eluent: isopropyl alcohol/*n*-hexane 1:99

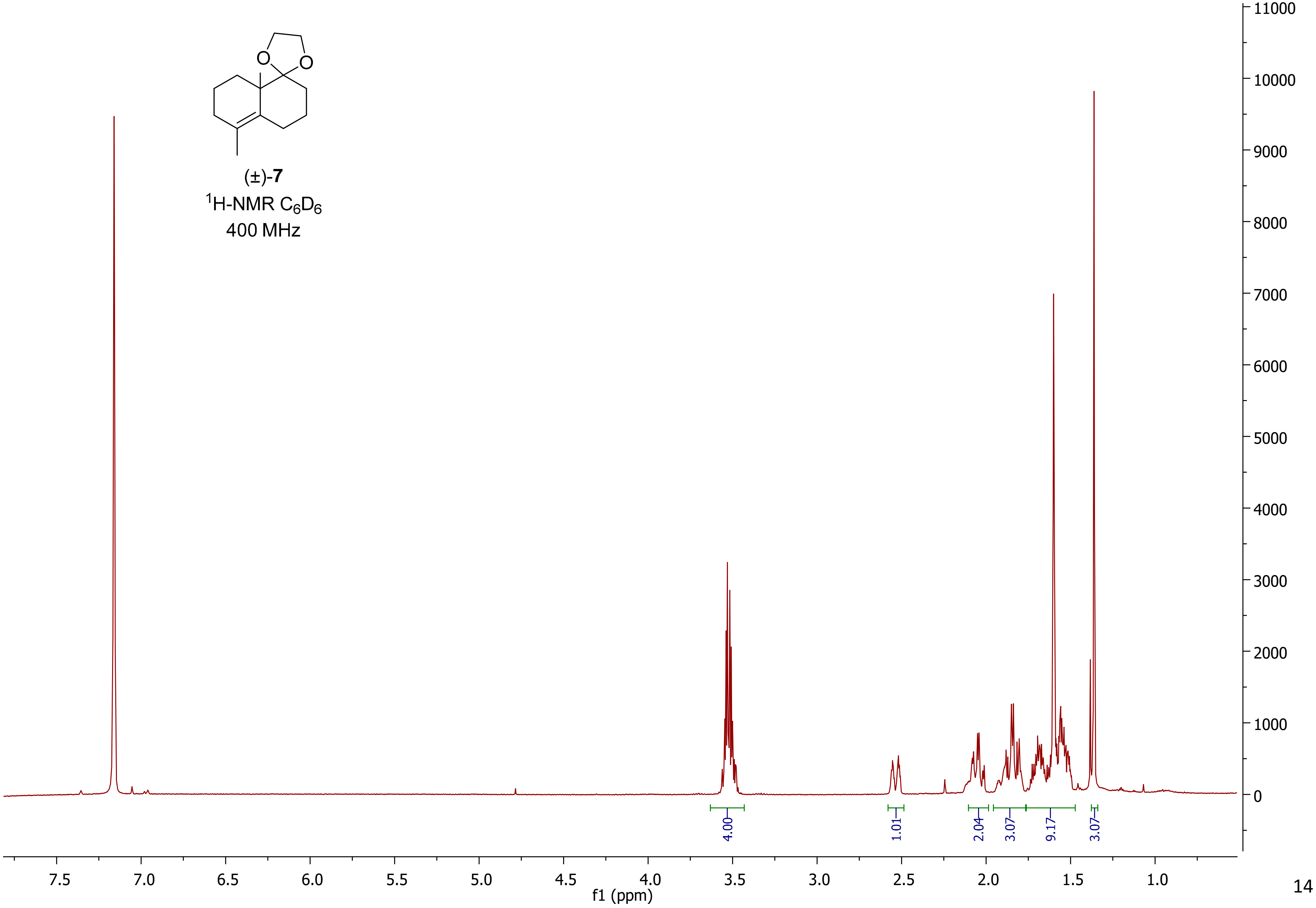
(±)-7
1H-NMR C6D6
400 MHz
4.00
1.01
2.04
3.07
9.17
3.07
f1 (ppm)

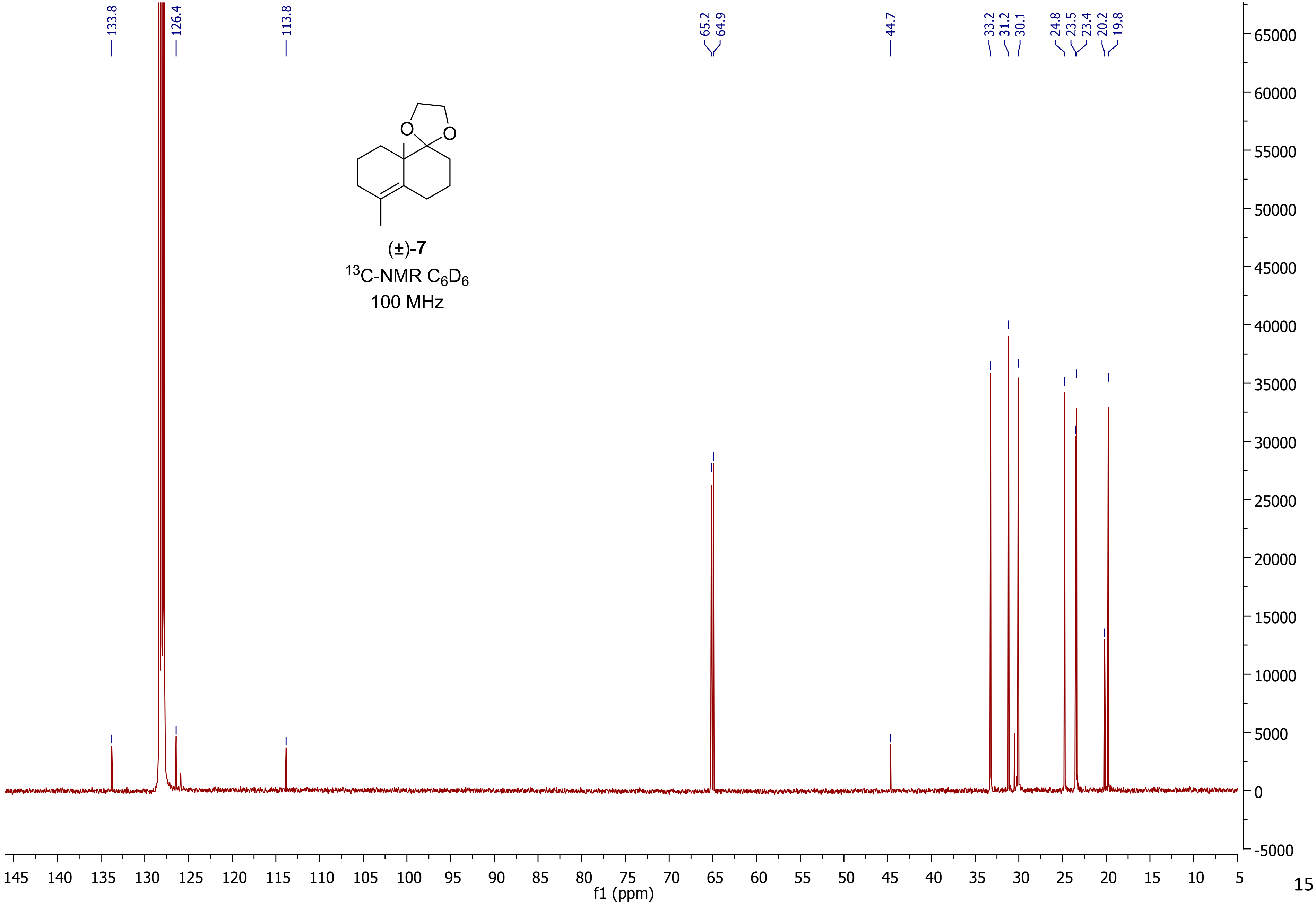
133.8
126.4
113.8
65.2
64.9
44.7
33.2
31.2
30.1
24.8
23.5
23.4
20.2
19.8
(±)-7
^{13}C-NMR C_6D_6
100 MHz
f1 (ppm)

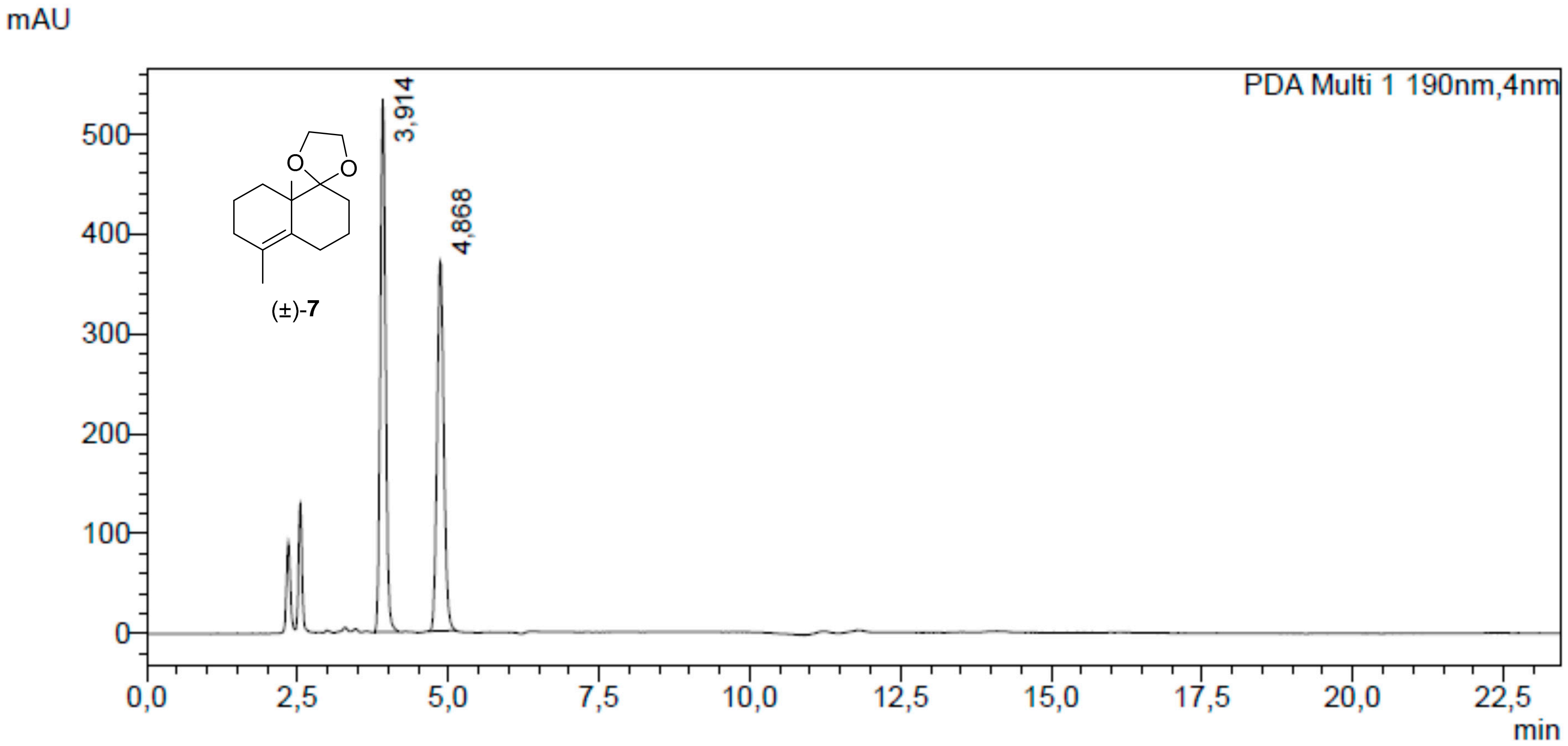

Column: Phenomenex Lux 3U Cellulose-4 4.60 x 150 mm;

Flow rate: 0.8 mL/min

Eluent: *n*-hexane

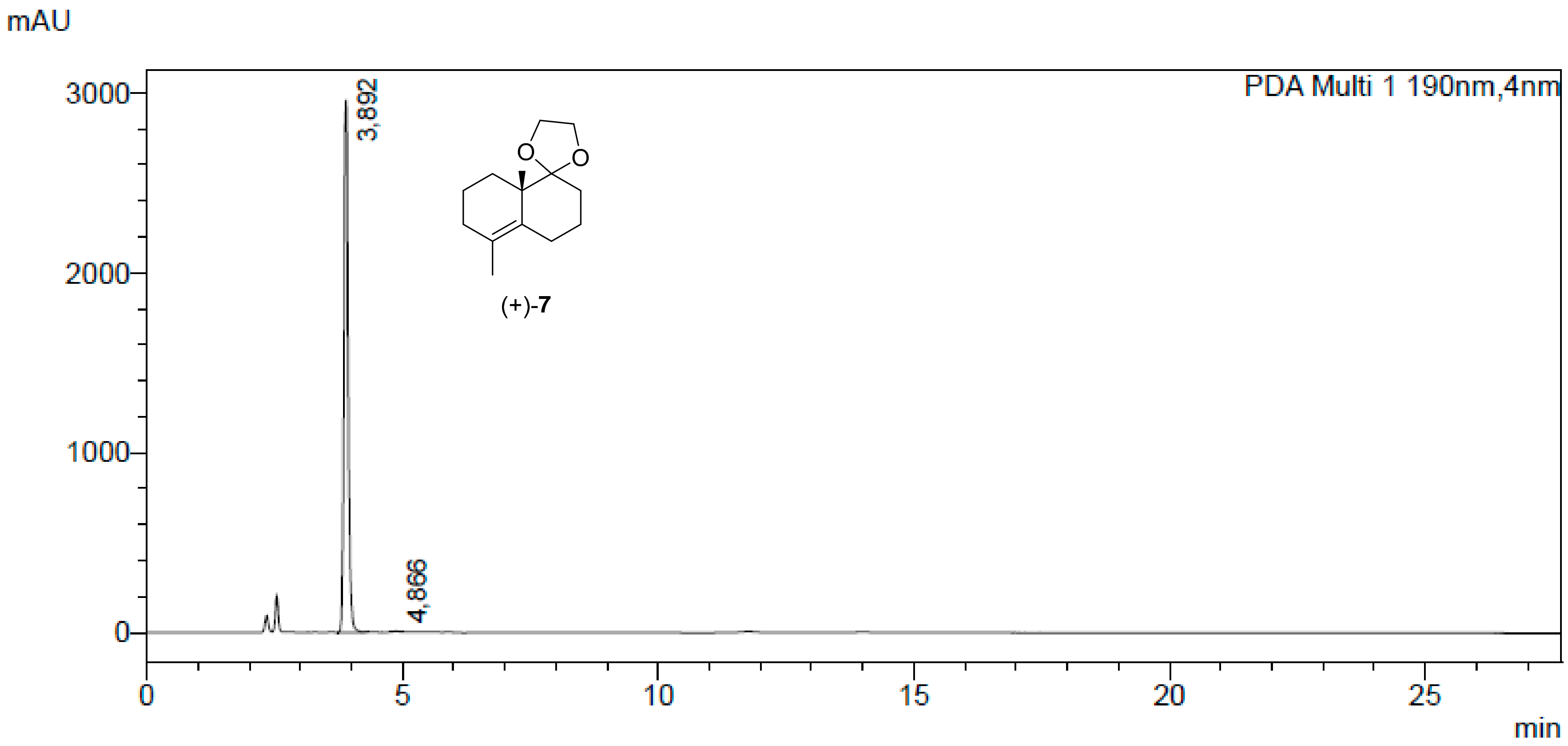

Column: Phenomenex Lux 3U Cellulose-4 4.60 x 150 mm;

Flow rate: 0.8 mL/min

Eluent: *n*-hexane
